# E-selectin in vascular pathophysiology

**DOI:** 10.3389/fimmu.2024.1401399

**Published:** 2024-07-19

**Authors:** Jinjin Zhang, Shengshi Huang, Zhiying Zhu, Alex Gatt, Ju Liu

**Affiliations:** ^1^ Department of Laboratory Medicine, Shandong Provincial Qianfoshan Hospital, Shandong University, Jinan, China; ^2^ Medical Research Center, Shandong Provincial Qianfoshan Hospital, Shandong University, Jinan, China; ^3^ Institute of Microvascular Medicine, Medical Research Center, The First Affiliated Hospital of Shandong First Medical University & Shandong Provincial Qianfoshan Hospital, Jinan, China; ^4^ Shandong Provincial Key Medical and Health Laboratory of Translational Medicine in Microvascular Aging, Jinan, China; ^5^ Department of Pathology, Faculty of Medicine and Surgery, University of Malta, Msida, Malta; ^6^ Haematology Laboratory, Department of Pathology, Mater Dei Hospital, Msida, Malta

**Keywords:** E-selectin, inflammation, oxidative stress, vascular diseases, therapeutic interventions

## Abstract

Selectins are a group of Ca^2+^-dependent, transmembrane type I glycoproteins which attract cell adhesion and migration. E-selectin is exclusively expressed in endothelial cells, and its expression is strongly enhanced upon activation by pro-inflammatory cytokines. The interaction of E-selectin with its ligands on circulating leukocytes captures and slows them down, further facilitating integrin activation, firm adhesion to endothelial cells and transmigration to tissues. Oxidative stress induces endothelial cell injury, leading to aberrant expression of E-selectin. In addition, the elevated level of E-selectin is positively related to high risk of inflammation. Dysregulation of E-selectin has been found in several pathological conditions including acute kidney injury (AKI), pulmonary diseases, hepatic pathology, Venous thromboembolism (VTE). Deletion of the E-selectin gene in mice somewhat ameliorates these complications. In this review, we describe the mechanisms regulating E-selectin expression, the interaction of E-selectin with its ligands, the E-selectin physiological and pathophysiological roles, and the therapeutical potential of targeting E-selectin.

## 1 Introduction

E-selectin (also known as ELAM1 [Endothelial Leukocyte Adhesion Molecule-1], CD62E, or LECAM-2), was first identified in the 1980s. It primarily regulates the adhesion and stable arrest of leukocytes to the endothelium in various disorders (1, 2). E-selectin remains largely inactive in resting endothelial cells, however, it is consistently expressed in response to inflammatory cytokines such as interleukin-1 (IL-1) bacterial lipopolysaccharide (LPS), viral infections, or tumor necrosis factor (TNF) (3–6). The constitutive expression of E-selectin and its regulatory mechanisms, including the involvement of key transcription factors, play a crucial role in controlling the baseline expression of E-selectin under normal physiological and pathological conditions. E-selectin facilitates the interaction of circulating leukocytes with vascular endothelium under inflammatory conditions. Additionally, it is important for the migration of hematopoietic stem cells, with its continuous presence on endothelial cells in hematopoietic tissues being crucial for the initial stage of their migration process. By cleaving membrane-bound E-selectin on the cell surface, the soluble form of E-selectin (sE-selectin) is generated (7). The levels of sE-selectin serve as surrogate markers of endothelial cell activation in response to inflammatory stimuli (8). However, the specific mechanism resulting in the cleavage that produces sE-selectin remains largely unexplored.

**Figure 1:**
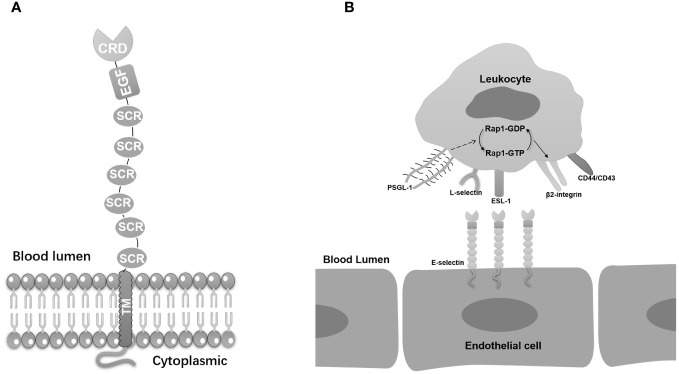
E-selectin’s basic molecular structure and its ligands. **(A)** E-selectin is composed of five distinct domains. The lectin-like domain, an epidermal growth factor (EGF)-like domain, and a short consensus repeat (SCR) domain. The lectin-like domain is located at the N-terminal region and is responsible for recognizing and binding specific carbohydrate ligands. The EGF-like domain, positioned in the middle, contributes to the overall structure and stability of E-selectin. The C-terminal SCR domain consists of multiple repeat units that facilitate protein-protein interactions and functions in cell adhesion processes. **(B)** E-selectin anchored on endothelial cells displays binding interactions with a diverse array of ligands on leucocytes, including PSGL-1, ESL-1, CD44, CD43, β2-integrins, and L-selectin. The combination of PSGL-1 on E-selectin activates Rap1, thereby inducing the activation of β2 integrin.

E-selectin belongs to the selectin family which is featured by homologous derived N-terminal determinants and all bind to similar fucosylated or sialylated glycan ligands (9). E-selectin interaction with its ligands contributes to acute and chronic inflammation, offering potential therapeutic avenues for addressing various diseases (10). The selectins, including P-selectin and L-selectin, share similar residues in spatial distributions with different performance in physiological activities. The E-selectin molecule comprises five unique components, including a lectin domain located at the amino terminus, which enables interaction with ligands, an epidermal growth factor (EGF)-like domain, complement regulatory-like domains, a transmembrane domain, and a cytoplasmic tail at the C-terminus. Each of these components influence the overall structure and function of E-selectin (**Figure 1A**) (11).

In this review, we will provide insights into molecular mechanism contributing to E-selectin expression, E-selectin and its cognate receptors, the expression of E-selectin under physiological and pathophysiological states and discuss the potential therapeutic targets towards E-selectin.

## 2 Physiological role of E-selectin

### 2.1 The function of E-selectin

E-selectin is upregulated in response to inflammatory cytokines stimulation (12). Upon white blood cell migration to inflammatory areas, the function of E-selectin extends beyond leukocyte chemotaxis and adhesion to include the regulation of inflammatory mediators and activation of inflammatory cells. On one hand, E-selectin binding to ligand molecules on the white blood cell surface activates downstream signaling pathways such as tyrosine kinase phosphorylation and protein kinase C. Activation of these pathways induces various cellular responses, including cytoskeletal rearrangement, changes in cell morphology, and regulation of cell functions, all crucial for leukocyte adhesion, migration, and inflammatory response development (13).

E-selectin’s physiological function is further exemplified by its facilitation of the adhesion of various cells, including neutrophils, monocytes, eosinophils, lymphocytes, and endothelial progenitor cells, primarily through integrin-mediated interactions (14–17). For instance, the endothelium facilitates the inflammatory process by regulating the transmigration of leukocytes through the involvement of E-selectin (18). Under normal physiological conditions, the egress of leukocytes from the bloodstream promotes pathogen elimination and tissue repair with assistance of E-selectin (19, 20). However, excessive leukocyte recruitment is deleterious, promoting acute and chronic inflammatory diseases (21). E-selectin serves as a key mediator of cellular interactions within the bone marrow endothelium. It plays a crucial role in the homing of hematopoietic stem cells and their progenitors to the bone marrow niche. On the bone marrow endothelium, E-selectin interacts with specific carbohydrate ligands present on the surface of these circulating cells, facilitating their rolling and tethering under physiological shear stress conditions. This interaction is a critical step in the multistep process of extravasation, ultimately leading to the selective recruitment and retention of hematopoietic cells within the bone marrow microenvironment (22).

In the field of diseases, soluble E-selectin (sE-selectin) has been implicated in rheumatoid arthritis, psoriasis, atherosclerosis, and cancer, regulating adhesive interactions between these cells and the endothelium (23–26). Elevated levels of sE-selectin have been observed in patients with inflammatory diseases and are indicative of a high-risk state (27–29). In ischemic mouse models, elevated plasma sE-selectin levels have been identified, with effects on intercellular cell adhesion molecule-1 (ICAM-1) expression and promotion of endothelial progenitor cell migration to ischemic tissues. This highlights the therapeutic potential of sE-selectin in enhancing neovascularization in ischemic organs (16).

Furthermore, sE-selectin is associated with cancer metastasis, with advanced stages of breast cancer showing increased serum E-selectin levels (30). It also negatively correlates with colorectal carcinoma survival rate and functions as a biomarker of colorectal carcinoma (31). E-selectin deficient mice are viable, fertile and of normal size (32). Upon stimulation by TNFα, there is a very small difference of leukocyte trafficking between E-selectin knockout mice and wild type mice. However, the velocity of the leukocyte recruitment process is significantly decreased in E-selectin deficient mice (32). Thus, E-selectin deficiency does not affect inflammatory responses in mice, but decelerates leukocyte rolling (33).

### 2.2 Transcriptional regulation of the *E-selectin* gene

The *E-selectin* gene transcription is increased upon the stimulation by inflammatory cytokines in endothelial cells. Sequence analysis showed that there are 3 NF-κB regulatory elements within the E-selectin promoter which regulates cytokine-induced expression located around the -170 site of *E-selectin* promoter (34). Activating transcription factor 2 (ATF2) transactivate *E-selectin* gene promoter activity through −166 binding site (35). High mobility group family of protein HMG I(Y) facilitates transcription factor ATF-2 binding to the *E-selectin* gene promoter. Meanwhile, HMG I(Y) promotes NF-κB regulatory elements binding to the promoter (36). Angiostatine K1–3 induced upregulation of *E-selectin* mRNA level, and K1–3 activated the promoter activity through AP1 (−195 site) and Ets-1(−90 site) (37). Runt-related transcription factor 1 (RUNX1) initiates the luciferase activity of at -320 site on E-selectin promoter and promotes gene transcription in endothelial cells (38). ERG, a member of the ETS (E twenty-six) family, plays a crucial role in regulating a variety of cellular processes such as cell proliferation (39), differentiation (40), development (41), and apoptosis (42). Its highly conserved DNA-binding domain, known as the ETS domain, allows it to interact with specific DNA sequences, termed ETS binding sites (43), thereby influencing the transcription of target genes. This interaction can either activate or repress gene transcription, significantly impacting the phenotype and behavior of cells. Notably, ERG suppresses E-selectin expression in HUVECs (Human Umbilical Vein Endothelial Cells) and elevation level of E-selectin expression was observed in embryos from Erg^−/−^ mice. In contrast, a higher level of E-selectin expression is observed in embryos from Erg^−/−^ mice, indicating a direct role of ERG in modulating E-selectin levels. Furthermore, ERG directly interacts with the *E-selectin* gene promoter, which may hinder its bioactivity. Interestingly, oxidative stress induction in endothelial cells leads to an increase in E-selectin expression and a simultaneous decrease in ERG expression, suggesting a complex interplay between these two factors in cellular regulation (44).

Oxidative stress enhances endothelial cell adhesiveness by increasing E-selectin expression and decreasing the transcription factor ERG, contributing to a prothrombotic state. ERG directly interacted with the -127 site of the *E-selectin* gene promoter, leading to a decrease in *E-selectin* gene activity in endothelial cells. Capture high-throughput chromosome conformation capture (CHi-C) indicated that no chromatin binding was caught on the *E-selectin* gene promoter (44). Krüppel-like factors 2 (KLF2) is a zinc finger transcription factor. KLF2 plays a pivotal role in mediating the anti-inflammatory function in endothelial cells. KLF2 overexpression significantly inhibited the mRNA and protein levels of E-selectin in human aortic endothelial cells (HAECs) (45). Transcription factor E2F-1 suppresses E-selectin expression in HAECs, and inhibits monocyte U937 cells adhesion to HAECs (46).

### 2.3 E-selectin ligands-regulated signaling pathways

Leukocytes in circulation interact with vascular endothelial cells via connections between endothelial E-selectin and glycan counter-receptors, known as E-selectin ligands, present on leukocytes. This initial binding leads to tethering, initiating the gradual rolling of leukocytes adhesion to the endothelium, a process characterized by velocities slower than the blood flow. The crucial slow rolling mediated by E-selectin enables close contact between monocytes and the inflamed endothelium (47).

The adhesion of selectins relies on lectin domain with ligands that carry glycans, specifically recognized the sialyl-Lewisx (sLex) and its isomer, sialyl-Lewisa (sLea) tetrasaccharide (48). E-selectin displays binding interactions with a diverse array of ligands. The most important E-selectin ligands are P-selectin glycoprotein ligand-1 (PSGL-1), CD44, and E-selectin ligand-1 (ESL-1). These interactions are pivotal for the recruitment of leukocytes to inflammatory sites, which is a critical aspect of the body’s defense against infection and injury (**Figure 1B**).

#### 2.3.1 PSGL-1

PSGL-1 is a ligand that interacts with selectin family members on leukocytes (49). L-selectin and P-selectin both bind to PSGL-1 at either the identical or closely adjacent sites near the N-terminus, while E-selectin seems to engage at least one additional site (50, 51). Apart from its involvement in facilitating leukocyte tethering and rolling, PSGL-1 also serves as a conduit for transmitting signals into both rolling leukocytes and those coated with platelets (52). Neutrophils in the process of rolling on P-selectin experience partial activation of integrin αLβ2, or lymphocyte-associated antigen-1 (LFA-1), resulting in a decrease in rolling velocities due to increased transient binding of LFA-1 to ICAM-1. Binding to E- or P-selectin promotes Syk-dependent elongation of LFA-1 (53, 54).

#### 2.3.2 CD44

CD44, a type I transmembrane glycoprotein found on a range of vertebrate cells, governs a multitude of cellular functions such as growth, survival, differentiation, and motility. Even though the cytoplasmic tail of CD44 does not possess inherent catalytic activity, it engages with Src family kinases (SFKs), Rho GTPase, Rho kinase, and protein kinase C (55). Neutrophils lacking CD44 demonstrate diminished adhesion to inflamed endothelium, leading to heightened rolling flux and velocities. CD44 exhibits binding to sE-selectin *in vitro* and works in collaborating with PSGL-1 to modulate rolling velocities and facilitate firm arrest *in vivo* (56–58).

#### 2.3.3 ESL-1

ESL-1 significantly reduces but not completely eliminating leukocytes rolling on E-selectin both *in vitro* and *in vivo* when PSGL-1 and CD44 are deleted (59, 60). Through knockdown experiments utilizing a short hairpin RNA approach, it has been observed that the interaction with a recombinant sE-selectin experiences a slight reduction in the absence of ESL-1, but is entirely abolished when both PSGL-1 and ESL-1 are knocked out (61).

#### 2.3.4 Other ligands interact with E-selectin

The intricate relationship between E-selectin and other molecules becomes more evident when considering versican, an aggregating proteoglycan in the extracellular matrix (ECM). Versican contributes to tissue integrity and cell signaling, with its carboxy terminus sharing sequence similarities with E-selectin’s EGF-like repeat (62, 63). These structural parallels may enable versican to directly engage with E-selectin, influencing leukocyte adhesion and migration, particularly during inflammation. Moreover, versican may affect the biding of E-selectin to PSGL-1 and CD44 (62, 64), potentially altering the dynamics of inflammatory responses.

## 3 Pathophysiological role of E-selectin in diseases

E-selectin modulates the inflammatory response present in many diseases. Oxidative stress is considered to be a direct product of inflammation. Below are the oxidative stress induced diseases with pathological implications of E-selectin expression.

### 3.1 Acute kidney injury

Oxidative stress contributes to the pathogenesis of acute kidney injury (AKI) by promoting cellular damage and inflammation (65). Kidney lesions induced by oxidative stress are accompanied by an elevated level of E-selectin. Indoxyl sulfate (IS) is one of the uremic toxins responsible for causing chronic kidney disease (CKD). IS, through the excessive generation of oxidative stress, damages vascular endothelium. Another study further confirmed that IS increases IL-1β-induced E-selectin expression in HUVECs. The molecular mechanism responsible for the interleukin-1β (IL-1β)-induced increase in E-selectin expression in IS-induced HUVECs involves phosphorylated MAPK signaling and the activation of NADPH oxidase/ROS (Reactive Oxygen Species) (66).

In addition, E-selectin is facilitated in the recruitment of neutrophil recruitment in AKI, which triggers inflammatory responding to ischemia-reperfusion. E-selectin on endothelial cells engages neutrophil ligands, initiating a cascade that activates integrins, enabling firm adhesion and tissue migration. Herter et al.’s research underscores the role of Phosphatidylinositol 3,4,5-trisphosphate-dependent Rac exchanger (P-Rex) in this process, showing its necessity for LFA-1 activation and neutrophil crawling (67). In AKI models, P-Rex1 deficiency lessens neutrophil infiltration and kidney damage, positioning E-selectin and P-Rex1 as potential therapeutic targets for mitigating AKI severity (67).

### 3.2 Pulmonary pathological processes

Oxidative stress promotes various pulmonary pathological progression (68–70). Asthma, identified as a persistent inflammatory condition, has been proposed as a potential risk element for endothelial dysfunction. An evident rise in E-selectin levels was also noted with increasing severity of asthma (71). In patients with Pulmonary Arterial Hypertension, soluble E-selectin was upregulated which might participate in local pulmonary recruitment of progenitor cell, causing endothelial activation (72). COVID-19 individuals showed high levels of sE-selectin in patients’ serum and plasma (73). All these facts raise the possibility that plasma E-selectin increased rapidly upon oxidative stress and leukocyte sequestration at sites of inflammation.

### 3.3 Hepatic pathologies

The liver, a pivotal metabolic organ, is the main organ in preserving immune and endocrine homeostasis. Oxidative stress significantly influences the advancement of hepatopathy (74–79). Hepatic failure resulting from ischemia/reperfusion (I/R) injury primarily stems from oxidative stress and inflammatory responses. The deterioration of the liver, coupled with abnormalities in pulmonary circulation, is attributed to oxidative stress and the release of inflammatory mediators during reperfusion (80). Emerging evidence suggests that E-selectin plays a critical role in the pathogenesis of hepatic disorders. Elevated levels of E-selectin have been detected in patients with non-alcoholic fatty liver disease (NAFLD) and alcohol-related liver disease (ARLD), making it a promising biomarker for disease monitoring and prognosis. Furthermore, experimental studies have shown that targeting E-selectin with specific antagonists can attenuate liver fibrosis in animal models, opening avenues for novel therapeutic strategies in chronic liver diseases (81, 82).

### 3.4 Venous thromboembolism

E-selectin serves as a critical regulator of thrombus formation and fibrin levels in a mouse model of venous thrombosis (83). Mice lacking E-selectin expression demonstrated decreased thrombus fibrin content, and mitigated vascular inflammation and fibrosis (84). In paroxysmal, persistent, and permanent atrial fibrillation, sE-selectin levels have been shown to be abnormal, indicating its underlying function in the prothrombotic state associated with the disease (85).

Similarly, analogous to pulmonary diseases, soluble E-selectin is linked to the pathological progression of hepatitis, liver cirrhosis, and hepatocellular carcinoma. Compared to healthy individuals, patients with chronic hepatitis and Child’s class A liver cirrhosis exhibit higher serum levels of sE-selectin. However, sE-selectin levels gradually decrease accompanied with the deterioration of liver cirrhosis. Furthermore, serum sE-selectin in hepatocellular carcinoma undergoes alterations corresponding to various indicators of cancer development (86). Notably, in leptospirosis, increased levels of sE-selectin have been observed, potentially interfering with immune cell recruitment and activation, underscoring the significance of endothelial activation in the disease process (87).

## 4 Therapeutic approaches targeting E-selectin

E-selectin plays a critical role in the adhesion of leukocytes to the endothelium during inflammation, a process facilitated by its interaction with specific carbohydrate ligands on the surface of these cells. This E-selectin/E-selectin ligand axis is a prime target for therapeutic intervention, aiming to disrupt the inflammatory cascade at its inception (10). The interaction mediated by E-selectin offers potential therapeutic avenues for addressing various diseases. Various exogenous inhibitors, such as carbohydrate molecules, carbohydrate mimics, small and large molecular mass non-carbohydrate compounds, peptides, antibodies targeting selectins, or nanoparticles, have been explored in this pursuit (88–91). Here we summarizes notable advancements and approaches in the development of pharmaceutical agents targeting E-selectin.

### 4.1 MicroRNAs

Numerous studies have focused on optimizing the therapeutic characteristics of miRNAs to repress E-selectin expression, thereby impeding the inflammatory process through the inhibition of JNK and NF-κB pathways. Small nucleotide miRNAs miR-31 and miR-146a (92) as well as si-E-selectin (93) successfully decreased the level of E-selectin. Nevertheless, the role of these microRNAs, known for their anti-inflammatory properties, remains largely unexplored in a metastatic context regarding their impact on the E-selectin-mediated extravasation of cancer cells. Besides, nucleotides may directly suppress the expression of E-selectin. Antisense oligonucleotides C-raf kinase apparently attenuate E-selectin regulated human colorectal carcinoma (CX-1 cells) adhesion. In addition, pretreatment nude mice with C-raf in nude mice inhibit E-selectin expression in nude mice prevent CX-1 cells hepatic metastasis (94).

### 4.2 Monoclonal antibodies

Accumulating evidence suggests that the infiltration of neutrophils into injured tissues can be effectively impeded by MAbs targeting E-selectin. Following intratracheal LPS injection in mice, the administration of anti-E-selectin monoclonal antibodies via the vein significantly inhibits neutrophil penetration into the bronchoalveolar space, achieving a reduction of 50–70% (95). The migration of leukocytes to ischemic cerebral tissue, coupled with an elevation in E-selectin expression, is particularly prominent in reperfused stroke. Mice subjected to treatment with anti–E-selectin exhibited a tendency to alleviate the side effects of ischemic injury, mitigating neurological deficits. This approach proves to be an appealing strategy for the prompt and effective treatment of cerebral ischemia (96).

### 4.3 Drugs directly targeting E-selectin

Sialyl Lewis X antigen is the physiological ligand of E-selectin (97). SLeX expressed on leukocytes or cancer cell surfaces interact with E-selectin, playing an anti-inflammatory function (98) and inhibiting cancer metastasis (99). SLeX serves as therapeutic agent as a potent E-selectin antagonist. However, the binding capacity of selectin–sLex is weak, hence, the primary ligand pattern was not an efficient drug delivery model. The synthesis of carbohydrate-based molecules is complicated and at a high cost (48, 100, 101). Analogues or mimetics of sLeX demonstrated higher binding affinity with E-selectin compared to natural sLeX in endothelial cells (48). Selectin mimetics are structurally based on sLex, the antagonism disrupt E-selectin-ligands interaction process, which provides therapeutic pathway towards inflammation (102). Non-toxic enzymatic like Fucosyltransferases decreased the production of E-selectin ligands sLex could inhibit inflammatory processes (103).

GMI-1271, a compact antagonist molecule that mimics the bioactive conformation of the sialyl-Lex/a carbohydrate ligand, acts as an inhibitor of E-selectin. This characteristic positions it as a promising candidate for potential therapeutic interventions and preventive measures against venous thrombosis (104). Additionally, GMI-1271 demonstrates promise as a therapeutic approach to reduce cancer mortality and impede cancer metastasis (105). Treatment human primary breast cancer cell line (CF1_T) with the fucosylation inhibitor 2-FF resulted in a significant reduction in E-selectin ligand expression, particularly sLeX/A. This led to a complete loss of CF1_T cell migration (106). Nonetheless, carbohydrate compounds face several drawbacks as drug candidates due to their susceptibility to enzymatic hydrolysis, diminished potency, and unfavorable pharmacokinetic properties.

Moreover, certain drugs have demonstrated direct effects on E-selectin expression, thereby influencing cancer progression. For example, cimetidine and amiloride have shown anti-cancer effect through directly block E-selectin expression in hepatocellular carcinoma (HCC). Indeed, the interaction of endothelial and HCC cells are interrupted (107). Cenicriviroc is CCR2 and CCR5 antagonist used in anti-HIV infection treatment. Cenicriviroc effectively inhibits monocyte trans-endothelial migration by disrupting monocyte-endothelial tethering through reduced E-selectin expression. Consequently, it serves as a therapeutic intervention to mitigate harmful monocyte trafficking (108).

Recently, significant progress has been made in generation of antagonists that exhibit a high affinity for targeting E-selectin. These advancements adhere to several key principles: 1) the incorporation of pharmacophoric groups into carbohydrate mimics; 2) the derivation of antagonist conformations from sLex; 3) the utilization of database screening employing the 3D pharmacophore of sLex, coupled with high throughput screening, resulting in the discovery of additional leads; and 4) achieving high bioavailability and binding affinity. The utilization of novel technology employing targeted bispecific molecules against E-selectin aids in assessing the anti-inflammatory therapeutic efficacy in a piglet model of enteritis (109). Additionally, a simplified nano-platform of dual prodrug mediated by the unique affinity between PSA (Prostate-Specific Antigen) and E-selectin binds nitric oxide and promotes vascular normalization (110).

## 5 Summary and conclusions

Endothelial cells uniquely express E-selectin, which initiates adhesion and recruitment of leukocytes, myeloid cells, and T-lymphocytes, thereby facilitating their extravasation into the surrounding tissues. E-selectin expression is induced by inflammatory stimulation and oxidative stresses, and the expression levels are associated with endothelial cell injury. Inhibition of E-selectin reduces thrombosis, vascular leakage and cancer metastasis, providing a potential avenue for new therapeutic interventions. Dysregulation of E-selectin has been found in several pathological conditions including AKI, pulmonary injury, hepatic failure and VTE. Deletion of the E-selectin gene in mice has been shown to mitigate complications associated with venous thrombosis, including decreased thrombus fibrin content and reduced vascular inflammation and fibrosis.

The prospect of sE-selectin as a biomarker is further supported by its measurable elevation in plasma, providing a non-invasive method of assessing endothelial status. However, the specificity and sensitivity of sE-selectin as a diagnostic or prognostic indicator may vary across different diseases, promoting further research to establish standardized thresholds and understand its pathophysiological role fully. Future researches should focus on clarifying the mechanisms regulating sE-selectin shedding and its interaction with other biomarkers to enhance our predictive capabilities. The development of targeted therapies that modulate sE-selectin levels may also present a novel avenue for therapeutic intervention in diseases characterized by endothelial dysfunction.

Collectively, these developments reinforce the potential of E-selectin as a promising therapeutic target against vascular diseases and other inflammatory conditions.

## Author contributions

JL: Writing – review & editing, Writing – original draft, Validation, Supervision, Conceptualization. JZ: Writing – review & editing, Writing – original draft, Validation, Conceptualization. SH: Writing – review & editing, Writing – original draft, Validation, Conceptualization. ZZ: Writing – review & editing. AG: Writing – review & editing.
